# An Analysis of Temperature-Dependent Timing Jitter Factors in the Structural Design of Complementary Metal-Oxide-Semiconductor Single-Photon Avalanche Detectors

**DOI:** 10.3390/s25020391

**Published:** 2025-01-10

**Authors:** Jau-Yang Wu, Yu-Wei Lu, Meng-Hsuan Liu, Tien-Ning Chang, Chun-Hsien Liu

**Affiliations:** 1 Department of Electrical Engineering, Yuan Ze University, Taoyuan 32003, Taiwan; 2 Institute of Electronics, National Yang Ming Chiao Tung University, Hsinchu 30010, Taiwan; wei19980929@gmail.com (Y.-W.L.); aa158467582952@gmail.com (M.-H.L.); emily7991e@gmail.com (T.-N.C.); terryliu225@gmail.com (C.-H.L.)

**Keywords:** CMOS SPAD, jitter, LiDAR

## Abstract

Single-Photon Avalanche Photodiodes (SPADs) are increasingly utilized in high-temperature-operated, high-performance Light Detection and Ranging (LiDAR) systems as well as in ultra-low-temperature-operated quantum science applications due to their high photon sensitivity and timing resolution. Consequently, the jitter value of SPADs at different temperatures plays a crucial role in LiDAR systems and Quantum Key Distribution (QKD) applications. However, limited studies have been conducted on this topic. In this study, we analyze the jitter characteristics of SPAD devices, focusing on the influence of device structures in two SPAD designs fabricated using the TSMC 18HV and TSMC 13HV processes. Using picosecond lasers with wavelengths ranging from ultraviolet (405 nm) to near-infrared (905 nm), we investigate the impact of different diffusion carrier types on jitter values and their temperature dependence across a range of 0 °C to 60 °C. Our results show that the jitter value of SPAD devices with low electric field regions varies significantly with temperature. This variation can be attributed to the higher temperature-dependent diffusion constant, as demonstrated by fitting the jitter diffusion tail with two diffusion time constants. In contrast, SPADs designed with modified electric field distributions exhibit smaller diffusion time constants and weaker temperature dependence, resulting in a much smaller temperature-dependent jitter value.

## 1. Introduction

The diode operates in Geiger mode, known as the GMAPD (Geiger Mode Avalanche Photodiode) or SPAD (Single-Photon Avalanche Diode). The high electric field in the active region enables high photon sensitivity and exceptional timing resolution. These physical advantages, combined with compatibility with CMOS technology, allow for the development of high-pixel arrays or the integration of multiple pixel outputs to achieve higher photon sensitivity, referred to as silicon photomultipliers (SiPMs) [1,2]. Such advancements are utilized in achieving longer-range LiDAR systems [3,4,5]. However, the timing resolution of SPADs, determined by their jitter value, directly affects the distance resolution and stability of LiDAR systems, as well as the transmission key rate in QKD applications [6,7]. LiDAR systems deployed in motor vehicles or Automated Guided Vehicles (AGVs) [8] must operate effectively across a wide temperature range while maintaining consistent distance resolution and accuracy. For QKD applications, low-temperature operation is often required to achieve ultra-low dark count rates in SPADs. Typically, automotive-grade 1 devices must operate within a temperature range of −40 °C to 125 °C and within a temperature range of −10 °C to +50 °C in the application of AGVs [9]. Therefore, the jitter value of SPADs across different temperatures is critical in both LiDAR and QKD applications.

Currently, even in mass-produced vehicle LiDAR systems, several non-ideal effects persist, such as secondary reflections, optical aberrations, and nonlinear optical characteristics of detectors. These issues have driven the development of various algorithms to improve distance resolution and reliability [10,11]. Digital signal processing technology, combined with CMOS technology, allows calibration algorithms to be integrated directly on the chip. This makes the digital output signal of SPADs particularly suitable for integration with DSP circuits to enhance the performance of LiDAR systems. Algorithms have been developed to address the non-ideal behavior of SPAD sensors, such as walk error effects, intensity-based aberrations, secondary reflections, and other issues [12,13]. However, the temperature-dependent behavior of SPAD sensors, particularly the temperature-dependent jitter value, has received less attention. This is critical, as variations in jitter values can significantly impact the distance resolution of LiDAR systems, yet there is little discussion about calibration algorithms addressing the temperature-dependent non-ideal effects in LiDAR modules. In some applications, SPAD devices are cooled to low temperatures to reduce noise, especially in SPADs with InGaAs/InP material [14]. Low-temperature environments are typically achieved using cryostats or cryogenic storage dewars. However, these solutions are bulky and consume significant power, making them unsuitable for mobile applications. A more practical solution for compact module applications is to use a thermoelectric cooler (TEC) co-packaged within a TO-cap package [15], which is the method chosen in this work for developing further applications after characteristic testing. TEC modules are also commonly used for maintaining specific chip temperatures to ensure consistent performance. While multi-stage TEC modules can achieve temperatures close to 200 K, they require substantial power. In this work, a single-stage TEC is used, which limits the achievable low temperature to approximately 275 K.

In our work, we compare two CMOS SPAD structures with similar designs, differing only in the custom doping concentration layer used to modify the electric field distribution. An analysis of our temperature-dependent timing jitter measurement results revealed that SPAD devices with weaker electric field regions exhibit significantly higher temperature-dependent timing jitter distributions. Furthermore, we examined the factors contributing to the variations in timing jitter under different temperatures and identified that these differences are influenced by the temperature-dependent diffusion coefficient of carriers, as described in ref. [16]. The diffusion coefficient of photocarriers shows a temperature dependence with an order of approximately negative 1.5, as explained in Ref. [16]. These insights could play a crucial role in developing calibration algorithms for SPAD sensors intended for high-temperature operational applications.

## 2. Materials and Methods

**Figure 1:**
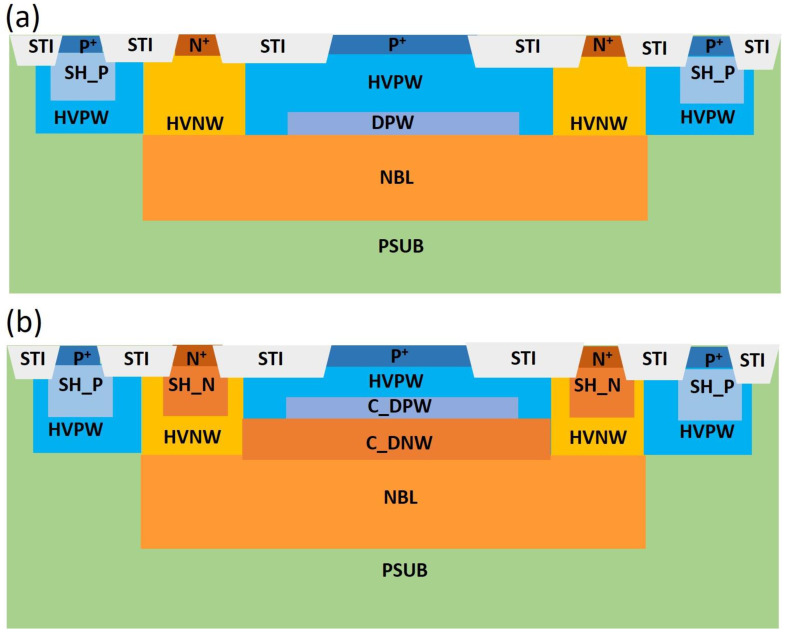
The cross-sectional views of the two measured SPAD device structures are shown: (**a**) a device fabricated using TSMC T18HV technology and (**b**) a device with a modified electric field distribution, fabricated using TSMC T13HV technology.

In our previous work, a p-type doping well, referred to as DPW, was introduced to reduce the breakdown voltage of the device. The active region consisted of a lower-doping-concentration p-type well (HVPW) and an n-type well (NBL). However, while the DPW layer effectively reduced the device’s breakdown voltage, it also caused a reduced electric field in the HVPW region, leading to poorer timing jitter performance compared to a device using only the HVPW and NBL junction. Detailed descriptions of this can be found in reference [17]. Subsequently, we introduced a customized n-type doping well (C_DNW) and a customized p-type doping well (C_DPW) to further reduce the breakdown voltage while modifying the electric field distribution in the HVPW region. These improvements were tested in two SPAD device structures fabricated using different CMOS technologies at various temperatures. The first SPAD device, referred to as SPAD1 in this discussion, was fabricated using the TSMC 0.18 μm HV process technology, with a breakdown voltage of 48 V, as shown in Figure 1a. The second SPAD device, referred to as SPAD2, was fabricated using the TSMC 0.13 μm HV process technology. SPAD2 incorporated two customized doping concentration processes based on our TCAD simulations, which are not supported by the standard TSMC 0.13 μm HV process technology. These customizations included the C_DPW and C_DNW layers, achieving a similar low breakdown voltage of approximately 16 V, as shown in Figure 1b.

**Figure 2:**
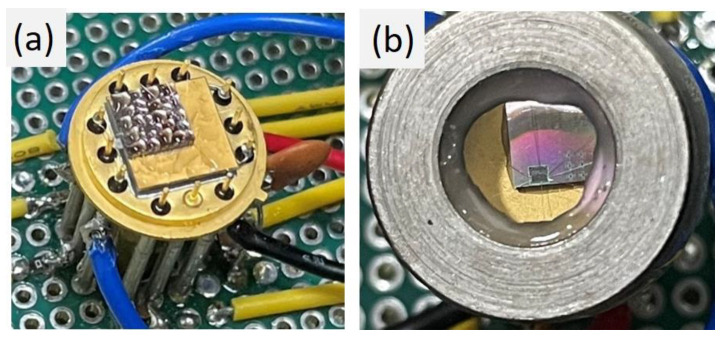
The SPAD chips are mounted in a TO-cap package on a TEC module (**a**), with the vacuum sealed using a cap (**b**).

The temperature characteristics of the two SPAD chips were measured by mounting them on a TEC within a TO-cap package. A vacuum was maintained to prevent moisture accumulation during low-temperature measurements, as shown in Figure 2a. Additionally, a cap with high-light-transmission glass was mounted, as shown in Figure 2b, allowing the SPAD parameters to be measured across various temperatures.

**Figure 3:**
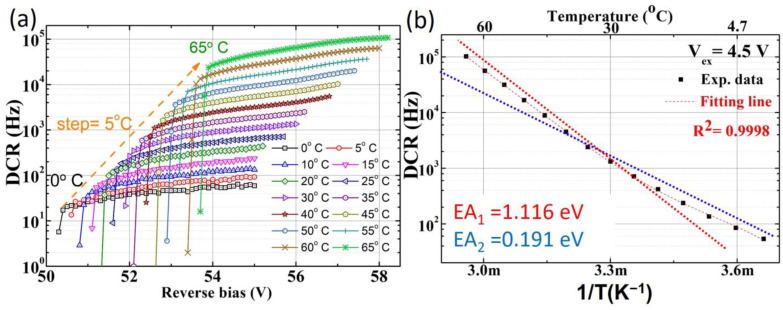
The temperature-dependent DCR is plotted at various bias voltages for SPAD1 (**a**), showing two distinct activation energies in the DCR plot at an excess bias voltage of 4.5 V (**b**).

The SPAD1 structure is designed with a breakdown voltage of 48 V and maintains a low DCR across different chips by fully utilizing the mask layers available in the standard CMOS TSMC 0.18 μm HV-BCD technology. Detailed characteristics can be found in reference [17]. The DCR decreases significantly when the operating temperature reaches 0 °C, as shown in Figure 3a. The activation energy can be extracted from the relationship between the DCR and temperature with the Arrhenius equation, as described in Equation (1) [18]. The analysis reveals two distinct activation energy values in the high- and low-temperature regions. The smaller activation energy (0.191 eV) in the low-temperature region indicates that tunneling effects dominate, leading to the low DCR observed at low temperatures for the SPAD1 chip, as shown in Figure 3b.

(1) $$DCR=A_{1}e^{\frac{-E_{a1}}{k_{B^{T}}}}$$

**Figure 4:**
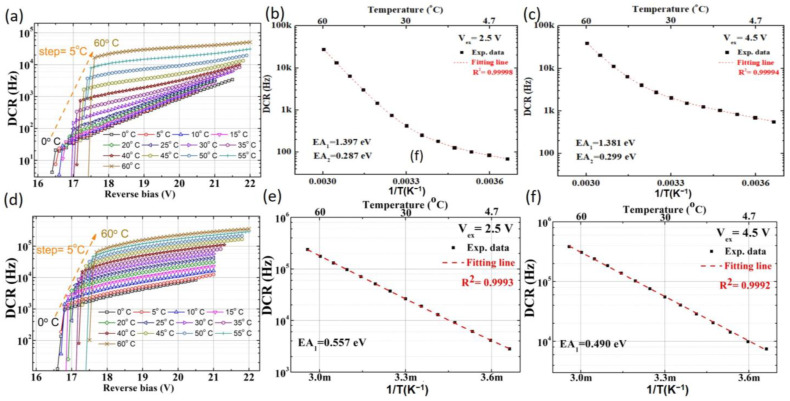
The temperature-dependent DCR is plotted at various bias voltages for the low-DCR SPAD2 chip. (**a**) Overall DCR trends, (**b**) showing two distinct activation energies at an excess bias voltage of 2.5 V and (**c**) at 4.5 V. Similarly, a temperature-dependent DCR is plotted for the high-DCR SPAD2 chip. (**d**) Overall DCR trends, (**e**) showing a single activation energy at an excess bias voltage of 2.5 V and (**f**) at 4.5 V.

However, compared to the uniform DCR distribution observed in SPAD1 across different chips, SPAD2 exhibits significant variation in DCR, with both high and low DCR performances evident from our measurements. Two customized layers are used in SPAD2 to modify the electric field, with process parameters designed based on our simulations. We believe the observed variations are due to suboptimal process parameters in the customized doping concentration process, such as the rapid thermal annealing process for recrystallization. This may lead to higher defect densities in some chips, resulting in a wide distribution range of DCR among SPAD2 chips. This is illustrated by the DCR of low-DCR chips in Figure 4a and high-DCR chips in Figure 4d. For low-DCR chips, two distinct slopes are observed in the DCR–temperature relationship under high and low excess bias voltage conditions. Correspondingly, an Arrhenius analysis reveals both high and low activation energies, as shown in Figure 4b,c. A lower activation energy (~0.29 eV) indicates that tunneling effects dominate the DCR at low temperatures. In contrast, for high-DCR chips, only a single slope is observed in the DCR–temperature relationship. A single activation energy is extracted from the Arrhenius analysis, as shown in Figure 4e,f. This indicates a simpler mechanism governing the DCR behavior in high-DCR SPAD2 chips.

**Figure 5:**
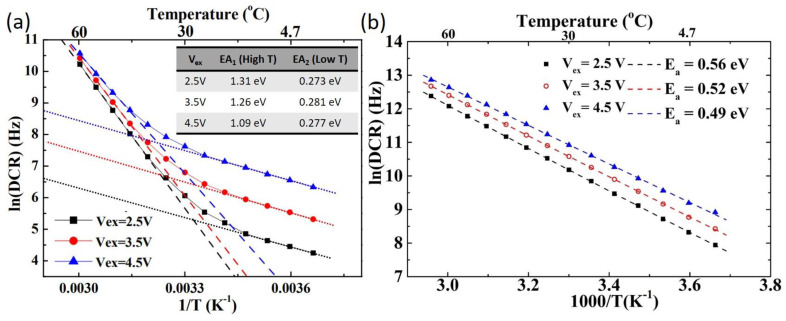
The Arrhenius plot for the low-DCR SPAD2 chip, along with the extracted two activation energies at three excess bias voltages, is shown in the inset table (**a**). Similarly, the Arrhenius plot for the high-DCR SPAD2 chip, displaying a single activation energy across three excess bias voltages, is presented in (**b**).

**Figure 6:**
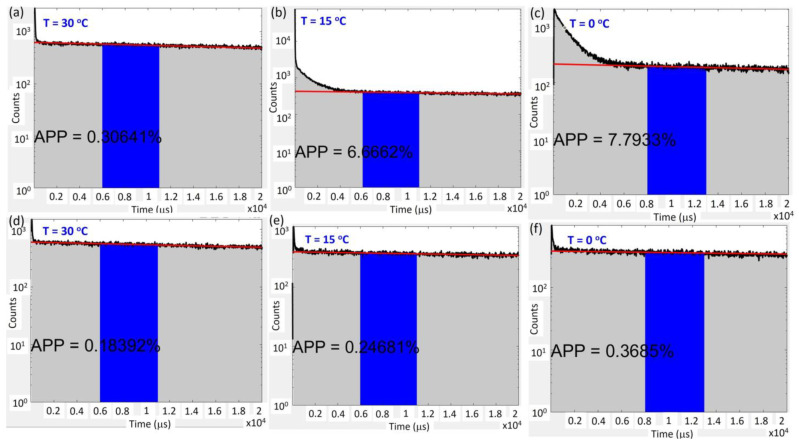
The high APP values observed at different temperatures during a short deadtime operation (50 ns) are plotted in (**a**–**c**). In contrast, significantly lower APP values are observed during a longer deadtime operation (100 ns) across various measurement temperatures are plotted in (**d**–**f**). The APP value is calculated as the sum of counts above the Poisson distribution fitting curve (plotted in red).

The Arrhenius plot of the low-DCR SPAD2 chip is shown in Figure 5a for further analysis. By fitting the DCR values at three excess bias conditions (2.5 V, 3.5 V, and 4.5 V) above the breakdown voltage with temperature, the activation energies (EAs) were extracted, as summarized in the inset table in Figure 5a. The small EA2 values observed across all excess bias voltages, with a consistent value of approximately 0.28 eV, indicate that the DCR at low temperatures is primarily caused by tunneling effects. In contrast, the activation energies extracted for the high-DCR chip at the same excess bias conditions are approximately half the silicon energy gap, as shown in Figure 5b, suggesting that the DCR is strongly influenced by the Shockley–Read–Hall (SRH) effect. We hypothesize that the higher defect density in high-DCR SPAD2 chips results from suboptimal customized process parameters. To validate this, the After-Pulsing Probability (APP) was measured using the Inter-Arrival Time (IAT) method [19,20], which confirmed that defects contribute to elevated DCR values. High APP values were observed at short deadtimes (50 ns) and low temperatures, as shown in Figure 6a–c. However, these high APP values disappeared during longer deadtime operations (100 ns) across various temperatures, as shown in Figure 6d–f. The presence of high APP values at short deadtimes and low temperatures in high-DCR SPAD2 chips explains the single activation energy observed at low temperatures, as shown in Figure 4e,f. A 100 ns deadtime was used for the temperature-dependent timing jitter measurement of the SPAD2 chip to prevent the APP from interfering with the analysis.

**Figure 7:**
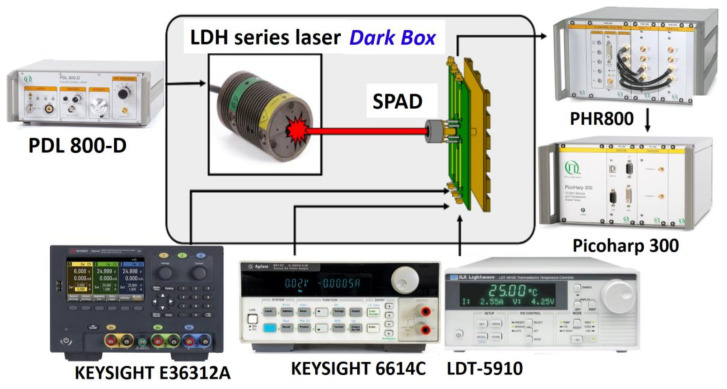
The setup for measuring jitter with temperature dependence using various wavelengths of picosecond lasers along with the diagram for the TCSPC method [22].

The setup for temperature-dependent jitter measurements is shown in Figure 7. Three picosecond lasers manufactured by PicoQuant Inc. (Berlin, Germany), with wavelengths of 405 nm, 780 nm, and 905 nm, are used for the analysis. These wavelengths, commonly applied in LiDAR and FLIM applications, span short to long wavelengths to investigate the mechanisms contributing to the temperature-dependent jitter values in different SPAD device structures. The SPAD chips are mounted on a TEC module equipped with a temperature sensor and housed in a TO-cap package with high-photon-transmission glass. Temperature control is automated using the temperature controller (Newport LDT-5910). The jitter values of the SPADs are measured using the Picoharp 300 (PicoQuant) with the Time-Correlated Single Photon Counting (TCSPC) method [21,22]. The TCSPC method is a statistical approach in which single-photon events are detected based on their arrival times, correlated with the pulse laser. This technique is commonly used to measure fluorescence decays in the time domain. To account for the SPAD’s dead time, a low pulse laser repetition rate (33 kHz) and low laser power are employed to prevent the occurrence of pile-up effects in the SPAD device. A long integration time (over 300 s in our measurements) is used to build the histogram of SPAD signals correlated with the pulse laser signals. The integration time is carefully chosen to ensure that the photon detection signal maintains a higher ratio relative to the DCR, achieving a sufficiently high signal-to-noise ratio for accurately analyzing the timing jitter behavior. Considering that the DCR of the SPAD device increases with a rising ambient temperature and excess bias voltage, and that the distribution of the DCR over time follows a Poisson distribution, the impact of this increase is relatively small because it is averaged across the 65,536 time bins of the TCSPC. Therefore, only a slight increase in laser power is required to raise the photon count probability, balancing the increased probability of DCR-triggered TCSPC events. This ensures that the peak value of the histogram remains significantly higher than the DCR level, maintaining a reliable analysis. Consequently, under various excess bias voltages and temperature measurement conditions, the timing jitter measurement integration time is carefully set to ensure that the photon count histogram tail can be clearly fitted and remains distinct from the average dark counts in each TCSPC time bin, even at the highest temperature and excess bias conditions.

## 3. Results

**Figure 8:**
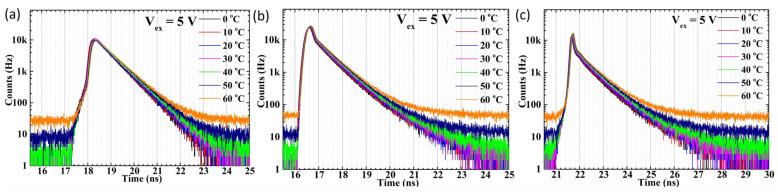
The plotted timing jitter distribution at various temperatures and 5 V excess bias voltages for the SPAD1 chip with 405 nm (**a**), 780 nm (**b**), and 905 nm (**c**) wavelengths.

**Figure 9:**
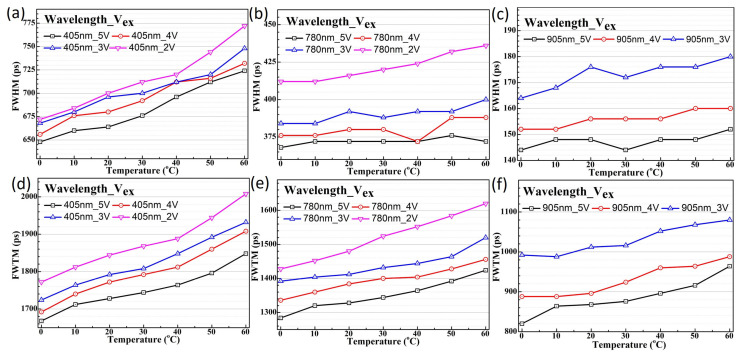
The plotted timing jitter FWHM values at various temperatures and excess bias voltages for the SPAD1 chip with 405 nm (**a**), 780 nm (**b**), and 905 nm (**c**) wavelengths and the FWTM values for 405 nm (**d**), 780 nm (**e**), and 905 nm (**f**). The excess voltage of 2 V is missing in the measurements at a wavelength of 905 nm.

The temperature-dependent jitter measurement results for the three different wavelengths reveal significantly different behaviors between the two SPAD devices. Two parameters are commonly used to quantify the timing performance of detectors in applications employing the TCSPC method, such as LiDAR and fluorescence lifetime imaging (FLIM). These are the full width at half maximum (FWHM) and the full width at tenth maximum (FWTM) values of the timing jitter histogram. The FWHM value represents the range within which photon detection events occur with a 50% probability, reflecting the timing uncertainty of the SPAD in the application. The FWTM value indicates the range within which over 90% of the total photon detection events occur. In the following discussion, we compare the FWHM and FWTM values of the two devices under temperature-dependent timing jitter measurements to further analyze and discuss the results of timing jitter performance. The temperature-dependent timing jitter profile for SPAD1 biased at 5 V is plotted in Figure 8. The FWHM values of the timing jitter distribution for SPAD1, biased at a 5 V excess bias voltage, are plotted against temperature for the 405 nm, 780 nm, and 905 nm picosecond lasers in Figure 9a–c, respectively. Notably, the main peak profiles show significant differences, with a larger temperature dependence being observed at shorter wavelengths compared to longer wavelengths, as shown in Figure 9. Under 405 nm laser illumination, the FWHM jitter distribution values are not only much larger but also exhibit stronger temperature dependence under all excess bias voltage conditions, as plotted in Figure 9a. Although smaller in magnitude, the FWHM values under longer wavelength laser illumination still display a noticeable temperature dependence. This behavior arises because the SPAD1 structure, as shown in Figure 1a, lacks an electric field in the HVPW layer. Consequently, photon carriers diffusing from the shallow absorption depth under 405 nm laser illumination result in a higher timing jitter distribution. The temperature-dependent timing tail caused by carrier diffusion is particularly evident in the FWTM values, plotted in Figure 9d–f for 405 nm, 780 nm, and 905 nm wavelengths, respectively.

**Figure 10:**
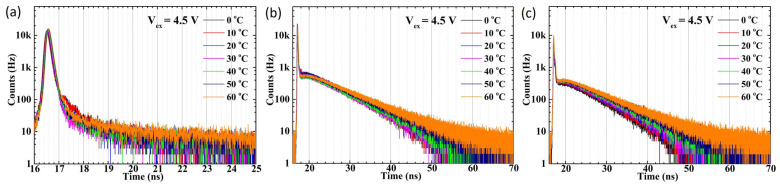
The plotted timing jitter distribution at various temperatures and 4.5 V excess bias voltages for the SPAD2 chip with 405 nm (**a**), 780 nm (**b**), and 905 nm (**c**) wavelengths.

**Figure 11:**
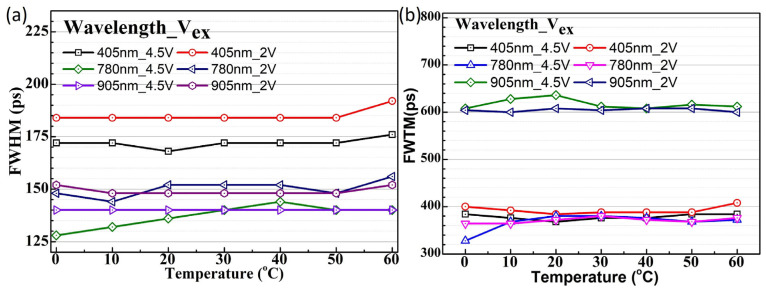
The plotted timing jitter FWHM values at various temperatures and excess bias voltages for the SPAD2 chip with 405 nm, 780 nm, and 905 nm (**a**) wavelengths and the FWTM values for 405 nm, 780 nm, and 905 nm (**b**).

The temperature-dependent jitter value distribution of SPAD2 exhibits significantly different behavior compared to SPAD1. The main peak of SPAD2 shows a smaller jitter distribution regardless of low or high excess bias voltages, although a noticeable diffusion tail appears in the jitter distribution under long-wavelength illumination, as shown in Figure 10. As plotted in Figure 11, the FWHM values remain around 150 ps across all temperatures and excess bias conditions under various wavelength illuminations, as shown in Figure 11a. Similarly, the FWTM values exhibit no temperature dependence at any wavelength, as plotted in Figure 11b.

## 4. Discussion

The significant differences in timing jitter performance are primarily attributed to the distribution of the electric field and the position of photocarrier generation within the SPAD structure. At a wavelength of 405 nm, the photon absorption depth is very shallow, resulting in photocarriers being concentrated near the surface. These carriers must diffuse through the HVPW layer, which has a weak electric field, to reach the avalanche region. This process leads to a broad peak in the timing jitter distribution and a small tail. In contrast, at wavelengths of 780 nm and 905 nm, photons penetrate deeper, even reaching the avalanche region. This deeper penetration results in many photocarriers being generated within the high electric field, causing a narrower peak in the timing jitter distribution. However, some electron carriers diffuse from regions deeper than the avalanche region, creating a wider diffusion tail.

The temperature-dependent timing jitter performance is significantly influenced by carrier diffusion, as the diffusion coefficient decreases with a rising temperature [23]. These effects are clearly illustrated in the plotted timing jitter figures. The diffusion coefficients of electrons and holes in silicon vary significantly, as described in Equation (2). Carriers from different doping layers contribute to distinct diffusion tails in the timing jitter distribution depending on the photon absorption depth and the electric field distribution.

(2) $$\tau =\frac{w_{n,p}^{2}}{\pi D_{h,e}}$$

To analyze the causes of the significant differences between the two SPAD device structures and address the carrier diffusion effects, which are not as prominent in the SPAD2 device due to the custom doping layer used to modify the electric field distribution in the HVPW, we applied a fitting approach. Using one or two time constants in the exponential decay equation, representing either a single carrier or the combined diffusion effects of both electrons and holes, as described in Equation (3), we fit the diffusion tails of the timing jitter curves for the two SPAD device structures, as shown in Figure 1.

(3) y=A1e−xτ1+A2e−xτ2

**Figure 12:**
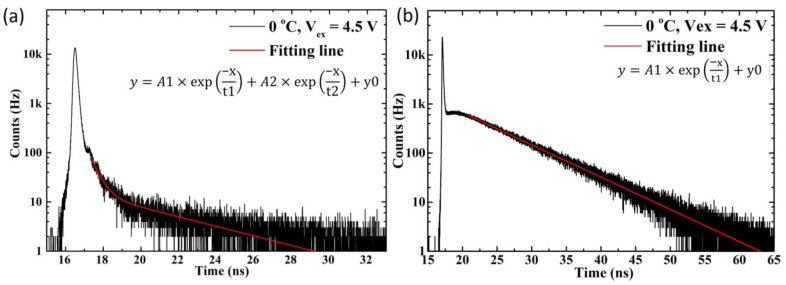
The plotted timing jitter distribution with the two-time constant fitting curve for the 405 nm laser illumination of SPAD2 (**a**) and the single-time constant fitting curve for the 780 nm laser illumination of SPAD2 (**b**).

For example, two time constants were used to fit the timing jitter distribution of SPAD2 under 405 nm laser illumination. In contrast, only one time constant was needed to fit the results under 780 nm and 905 nm laser illumination, as shown in Figure 12.

**Figure 13:**
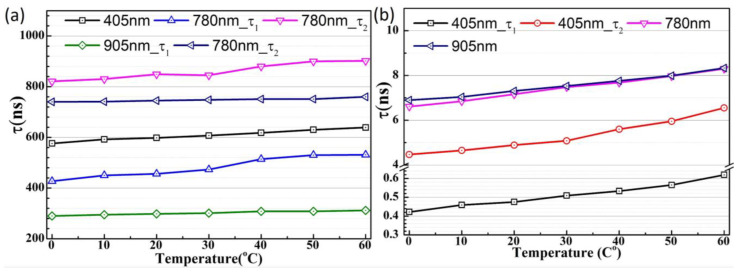
The time constants plotted with temperature for SPAD1 with various wavelengths (**a**) and for SPAD2 (**b**).

From the fitted time constant plot, it can be observed that the SPAD1 device exhibits significantly larger diffusion time values with a strong temperature dependence, as shown in Figure 13a. This behavior can be explained by the electric field distribution in the SPAD1 device, which lacks a sufficient electric field presence in the HVPW layer. In contrast, the SPAD2 device shows much smaller diffusion time constants with minimal temperature dependence in its timing jitter characteristics, as depicted in Figure 13b. This improvement can be attributed to the modified electric field distribution in the HVPW layer achieved through the customized doping layer. Based on the extracted time constant results, we conclude that the temperature-dependent timing jitter variance primarily originates from carrier diffusion behavior, with single or combined carrier diffusion triggering avalanche events.

## 5. Conclusions

The high timing resolution characteristic of SPAD devices makes them widely applicable in high-performance LiDAR systems and quantum science. The timing jitter of SPAD devices contributes to detection range uncertainty in LiDAR systems and limits the transmission key rate in quantum key distribution (QKD). However, the temperature dependence of timing jitter has been rarely discussed. In this work, we analyze two SPAD devices with similar structures, where one device incorporates a customized doping layer to modify the electric field distribution. Based on our measurement results, the modified electric field suppresses carrier diffusion, resulting in lower temperature-dependent timing jitter. In contrast, the unmodified device exhibits significantly higher temperature-dependent timing jitter values.

Our findings demonstrate that carrier diffusion plays a critical role in the temperature dependence of timing jitter, particularly contributing to the jitter distribution tail. These insights provide a foundation for developing temperature-dependent calibration algorithms to improve timing resolution performance.

## Data Availability

Data are contained within the article.

## References

[1] Villa F., Severini F., Madonini F., Zappa F. (2021). SPADs and SiPMs Arrays for Long-Range High-Speed Light Detection and Ranging (LiDAR). Sensors.

[2] Yang R., Tang Y., Fu Z., Qiu J., Liu K. (2022). A method of range walk error correction in SiPM LiDAR with photon threshold detection. Photonics.

[3] Incoronato A., Locatelli M., Zappa F. (2021). Statistical Modelling of SPADs for Time-of-Flight LiDAR. Sensors.

[4] Scholes S., Mora-Martín G., Zhu F., Gyongy I., Soan P., Leach J. (2023). Fundamental limits to depth imaging with single-photon detector array sensors. Sci. Rep..

[5] Wang Z., Yang X., Tian N., Liu M., Cai Z., Feng P., Dou R., Yu S., Wu N., Liu J. (2024). A 64 × 128 3D-Stacked SPAD Image Sensor for Low-Light Imaging. Sensors.

[6] Madonini F., Severini F., Zappa F., Villa F. (2021). Single photon avalanche diode arrays for quantum imaging and microscopy. Adv. Quantum Technol..

[7] Keshavarzian P., Ramu K., Tang D., Weill C., Gramuglia F., Tan S.S., Tng M., Lim L., Quek E., Mandich D. (2023). A 3.3-Gb/s SPAD-based quantum random number generator. IEEE J. Solid-State Circuits.

[8] Ito S., Hiratsuka S., Ohta M., Matsubara H., Ogawa M. (2018). Small imaging depth LIDAR and DCNN-based localization for automated guided vehicle. Sensors.

[9] https://hokuyo-usa.com/application/files/6316/8451/4574/Hokuyo_-_UST-05LX_-Final.pdf.

[10] Levinson J., Askeland J., Becker J., Dolson J., Held D., Kammel S., Kolter J.Z., Langer D., Pink O., Pratt V. Towards fully autonomous driving: Systems and algorithms. In Proceedings of the 2011 IEEE intelligent vehicles symposium (IV), Baden-Baden, Germany, 5–9 June 2011; IEEE: Piscataway, NJ, USA, 2011.

[11] Lambert J., Carballo A., Cano A.M., Narksri P., Wong D., Takeuchi E., Takeda K. (2020). Performance analysis of 10 models of 3D LiDARs for automated driving. IEEE Access.

[12] Chai Z., Sun Y., Xiong Z. (2018). A novel method for lidar camera calibration by plane fitting. Proceedings of the 2018 IEEE/ASME International Conference on Advanced Intelligent Mechatronics (AIM).

[13] Wang X., Ma R., Li D., Zheng H., Liu M., Zhu Z. (2020). A low walk error analog front-end circuit with intensity compensation for direct ToF LiDAR. IEEE Trans. Circuits Syst. I Regul. Pap..

[14] Lee Y.-S., Chen T.-Y., Chen Y.-J., Kan W.-H., Liu X.-W., Shi J.-W. (2024). Photon-Number-Resolving Detection with Highly Efficient InGaAs/InAlAs Single-Photon Avalanche Diode. Photonics.

[15] Itzler M.A., Entwistle M., Owens M., Patel K., Jiang X., Slomkowski K., Rangwala S., Zalud P.F., Senko T., Tower J. (2011). Comparison of 32 × 128 and 32 × 32 Geiger-mode APD FPAs for single photon 3D LADAR imaging. Adv. Photon Count. Tech. V.

[16] Batista J., Mandelis A., Shaughnessy D. (2003). Temperature dependence of carrier mobility in Si wafers measured by infrared photocarrier radiometry. Appl. Phys. Lett..

[17] Huang L.D., Wu J.Y., Wang J.P., Tsai C.M., Huang Y.H., Wu D.R., Lin S.D. (2017). Single-photon avalanche diodes in 0.18-μm high-voltage CMOS technology. Opt. Express.

[18] Xu H., Pancheri L., Betta G.F.D., Stoppa D. (2017). Design and characterization of a p+/n-well SPAD array in 150 nm CMOS process. Opt. Express.

[19] Wahl M., Rahn H.-J., Gregor I., Erdmann R., Enderlein J. (2007). Dead-time optimized time-correlated photon counting instrument with synchronized, independent timing channels. Rev. Sci. Instrum..

[20] Hofbauer M., Steindl B., Zimmermann H. (2018). Temperature Dependence of Dark Count Rate and After Pulsing of a Single-Photon Avalanche Diode with an Integrated Active Quenching Circuit in 0.35 μm CMOS. J. Sens..

[21] Becker W. (2023). The bh TCSPC Handbook.

[22] Wahl M. (2004). Technical Note on TTTR.

[23] Brunetti R., Jacoboni C., Nava F., Reggiani L., Bosman G., Zijlstra R.J.J. (1981). Diffusion coefficient of electrons in silicon. J. Appl. Phys..

